# Obituary

**Published:** 1896-04

**Authors:** 


### Obituary.

Dr. R. H. Wilson died, after several weeks of lingering illness, of la grippe, at his home on Chestnut street, Louisville, Ky., on Sunday, March 22, 1896. He came to Louisville more than forty years ago, in his young manhood, and conducted a very lucrative practice. He reared a family of sons, two or three of whom graduated in dentistry. He materially assisted many other young men in the details of mechanical dentistry, in which he was counted to be an expert and master. He was a helpful, obliging man, always ready to do a brother dentist any service he might need. In the department of mechanical work for the past fifty years, Dr. Wilson did more than any one who has practiced in Louisville. He had not the advantages of early and careful education, but his manly heart to do the best and a conscience that was void of offence gave him a wide circle of friends, who now mourn his loss. By his integrity, uprightness and Christian life, he was esteemed as a valuable citizen, as was shown by the gathering of aged people who thronged his funeral obsequies. At the ripe age of seventy-two, full of good deeds, beloved by friends and interesting children of mature age, Dr. R. H. Wilson passes away into rest. W. F. M.

